# Is It Safe to Operate without Frozen Section Biopsies in Short-Segment Hirschsprung’s Disease? An Overview of 60 Cases

**DOI:** 10.3390/pediatric16030045

**Published:** 2024-06-25

**Authors:** Isber Ademaj, Nexhmi Hyseni, Naser Gjonbalaj

**Affiliations:** 1 Department of Pediatric Surgery, University Clinical Center of Kosovo, 10000 Pristina, Kosovo; nexhmi_h@yahoo.com; 2 Department of Radiology, University Clinical Center of Kosovo, 10000 Pristina, Kosovo

**Keywords:** short-segment Hirschsprung’s disease, frozen section biopsy, Surgeon’s intraoperative judgment

## Abstract

**Background:** Advancements in surgical management in a single-stage procedure made intraoperative frozen section biopsies critical for determining of level of resection to avoid the potential risk of leaving a retained aganglionic segment. However, in most low-income countries, due to the lack of this facility, the surgeon’s intraoperative judgment is used for the determination of the resection level. **Objective:** This study aims to evaluate the accuracy of determining the level of bowel resection in short-segment Hirschsprung’s disease based on macroscopic changes. **Materials and methods:** Intraoperative macroscopic evaluations were assessed using postoperative microscopic findings to determine whether the surgeons’ intraoperative judgments were accurate in determining the level of bowel resection in 60 cases of operated short-segment Hirschsprung’s disease. In addition, Pearson’s correlation coefficient was used to determine whether the sensitivity and specificity of both methods were significantly correlated. **Results:** The microscopic results showed that the level of resection based on the macroscopic evaluation was performed in normally ganglionated segment in cases of short-segment Hirschsprung’s disease. **Conclusions:** Macroscopic intraoperative assessment by an experienced surgeon is highly accurate method of determining the level of bowel resection in short-segment HSCR.

## 1. Introduction

Hirschsprung’s disease (HSCR) is characterized by the absence of ganglion cells in the distal part of the digestive tube, which is manifested by functional obstruction in the a-ganglionic segment (AGS). The AGS due to non-relaxation of the musculature is narrow in caliber, which proximally continues into the hypo ganglionic segment, forming a funnel shape typically marked by a sudden increase in the width above which the dilated normoganglionic segment (NGS) can be seen [1]. The gold standard for the diagnosis of HSCR is currently the histopathological diagnosis performed on samples obtained mainly via trans-rectal biopsy. Microscopic examination of these samples only detects rectal aganglionosis and does not provide data on the length of the AGS and transition zone (TZ), which must be resected during the definitive treatment. During surgical treatment, determining the level of resection in the NGS is crucial in order to avoid eventual obstructive postoperative complications related to TZ and AGS [2]. Microscopic evaluation of samples provided by a frozen section biopsy (FSB) is necessary to precisely determine the level of resection. However, it requires collaboration with the pathologist, resulting in additional costs and significant waiting times However, these morphological changes in bowel caliber become more clearly differentiated macroscopically after the first month of life, especially after the third month of life. They differentiate into the narrow AGS, the hypoganglionic funnel-shaped (TZ) segment, and the dilated NGS, which are also detected, in contrast enemas, typically after the first month of life [3]. The goal of surgical management in HSCR is to resect both the AGS and the TZ [2]. The TZ is characterized by hypertrophic nerve fibers and a decreased density of ganglion (submucosal) cells, a condition described as hypoganglionosis. However, analyzing TZ from FSB samples is prone to inaccuracy [4,5]. Inaccuracies in determining the level of resection in the NGS and incorporating the TZ during the anastomosis can lead to complications, mainly constipation and Hirschsprung-associated enterocolitis (HAEC) after the operation [6]. The current intraoperative HSCR treatment algorithm involves the macroscopic identification (surgeon’s intraoperative judgment) of the TZ, above which an FSB is obtained in the dilated segment to determine the level of resection in the NGS. However, some pediatric surgeons, against the general agreement for mandatory histopathological diagnosis, operate on cases with classic characteristics of HSCR, relying only on contrast enema and clinical characteristics without preoperative histopathological confirmation. They perform HP diagnosis and determine the level of resection only intraoperatively through FSB [7,8]. In some hospitals, due to the lack of facilities for FSB, surgeons rely on contrast enemas to predict the AGS preoperatively, in some cases opening the stoma and taking biopsies in the suspected segment, known as “colonic mapping” is performed [9,10]. With this retrospective and prospective study, we report our two-decade experience of operating without FSB and the accuracy of determining the level of bowel resection based solely on macroscopic changes.

## 2. Materials and Methods

Between 2000 and 2020, in our clinic, were operated 60 patients diagnosed with short-segment HSCR. Diagnosis was confirmed from samples obtained via full-thickness biopsy (FTB), taken 2.5 to 4 centimeters above the dentate line, at least 2 months before definitive surgical treatment. In all cases, a contrast enema was performed at least one month before the surgical procedure. The length of the AGS ranged from 6 cm above the dentate line to the lower third of the descending colon. Three cases with total colonic aganglionosis (TCA) were not included in this study, and no case was diagnosed as ultra-short-segment HSCR. The age of the patients at the time of operation ranged from 9 months to 8 years. During the definitive surgical management, the level of resection was determined based solely on a macroscopic assessment. The objective criterion used for the macroscopic determination of TZ during the morphological assessment was the site of apparent change in caliber, typically marked proximally to the narrowed AGS. In all cases, resections were performed proximally to the defined transition zone (TZ) in the normally ganglionated segment. In seven cases, resection was performed at 5 cm; in 18 cases, resection was performed at 7 cm; in 23 cases, resection was performed at 10 cm; and in 12 cases, resection was performed 15 cm proximally to the TZ in the normally ganglionated segment. The samples taken from the resected bowel after the operation were examined with a routine examination (with HE), while in the re-examination, they were examined using the IHC method with Calretinin for ganglion cells and with S100 for nerve fibers. Two independent pathologists with experience in the diagnosis of HSCR carried out the re-examination of the samples. From the proximal margins of the resections, a transverse section with a width of 0.5–0.7 cm was taken from each resection to confirm the normally ganglionated area in the entire circumference of the resections. Data from the macroscopic intraoperative evaluation and microscopic hematoxylin and eosin (HE) and IHC postoperative findings were compared using the Kappa statistic to determine whether macroscopic assessment is an accurate method in predicting the level for bowel resection. Pearson’s correlation coefficient was used to determine if the sensitivity and specificity of both methods were significantly correlated. Additionally, we compared the accuracy of the macroscopic and microscopic evaluations in the second and fifth centimeters above the TZ from the resected margin of 60 cases with short-segment HSCR in our study, aiming to assess the safety distance from the TZ to avoid unnecessary resection. This study was conducted in compliance with the principles of the Declaration of Helsinki and approved by the Ethical and Professional Committee. Since the study was morphological, retrospective, and unrelated to any additional clinical interventions, patient consent was not explicitly obtained. Additionally, informed consent was obtained at the time of surgery for any potential future research that might include tissue samples.

## 3. Results

**Figure 1:**
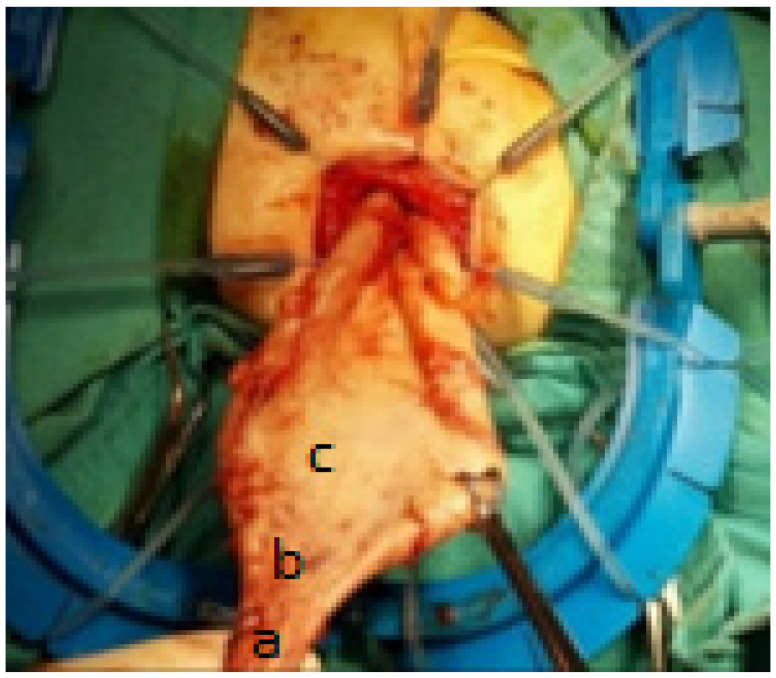
The intraoperative macroscopic assessment of the fully mobilized rectosigmoid segment using the transanal technique reveals a narrowed zone (a), a transition zone intermediate funnel-shaped in the rectosigmoid zone (b), and a dilated zone (c).

The intraoperative macroscopic assessment determined the level of resection with certainty, which was confirmed postoperatively by routine microscopic examination with HE, showing the presence of ganglion cells and non-hypertrophied nerve fibers in all resections from the fifth centimeter above the TZ, thus defining the level from the fifth centimeter above the TZ as a “safe level” (Figure 1).

From the proximal margin of the resections, NGS was found in 7 cases up to the fifth centimeter, in 18 cases up to the seventh centimeter, in 23 cases up to the tenth centimeter, and in 12 cases up to the fifteenth centimeter from the resection margin (Table 1).

IHC re-testing of the samples from the TZ and the dilated segment of the resection proved that the level of resection based on the macroscopic assessment was performed at the normally ganglionated level, resulting in complete concordance between the macroscopic evaluation and the microscopic examination regarding the level of resection (Kappa = 1) (Table 2). The presence of ganglion cells and non-hypertrophied nerve fibers was found in all cases from the fifth centimeter above the TZ.

Examination with HE and re-testing with IHC resulted in complete concordance regarding the analysis of samples from the TZ and the dilated segment. Also, interobserver concordance comparing IHC microscopic evaluations in samples from TZ and the dilated zone, performed by two different experienced pathologists and tested using Kappa statistics, showed perfect agreement between the two pathologists (Kappa = 1). We also compared the accuracy of the macroscopic and microscopic evaluations in the second and fifth centimeters above the TZ from the resections of 60 cases. There was a significant difference (Fisher’s exact test), showing that the microscopic evaluation was more reliable in the second centimeter, showing a 100% accuracy, than the macroscopic evaluation, which showed only 28.33% of predicted normoganglionosis to be true positives (*p* < 0.05). However, in the fifth centimeter comparison, there was no difference between the macroscopic and microscopic evaluation; both tests showed a normal ganglionic zone in all cases (*p* = 1.0) (Table 3).

Given that the macroscopic examination correctly identified the level of resection in the colon in all 60 cases of short-segment HSCR, resulting in a normally ganglionated segment of bowel showing ganglion cells in the microscopic examination, we found the sensitivity of the macroscopic examination to be 100% when the resection was performed from the fifth centimeter above the transition zone TZ. Since there were no AGSs misclassified as normally ganglionated based on the macroscopic examination, the specificity was also 100%. To determine if the sensitivity and specificity of the macroscopic and microscopic examinations were significantly correlated, we used Pearson’s correlation coefficient, which indicated a perfect positive correlation between examinations (r = 1.0). However, due to the perfect correlation and the small sample size, it is not possible to determine whether the sensitivity and specificity are significantly different. Since the correlation was perfect (r = 1), the *p*-value was zero, indicating a statistically significant correlation. The correlation between the sensitivity and specificity was statistically significant at *p* < 0.001. This means that the observed correlation was very unlikely to be due to chance.

## 4. Discussion

In HSCR, the absence of ganglion cells observed at the microscopic level is consistently accompanied by morphological changes observed macroscopically, typically distinguished after the third month of life as a narrowed AGS, a funnel-shaped hypoganglionic segment (TZ), and a dilated NGS. Surgical management of HSCR involves resection of the AGS and TZ, determined based on contrast enema findings preoperatively [11]. However, significant differences have been described between TZs determined based on contrast enemas and those found intraoperatively using FSB [12]. Frongia et al. reported a 94.4% concordance of contrast enemas with the histologically assessed TZs in cases with rectosigmoid HSCR, but only 50% in those with long-segment HSCR [3]. Also, contrast enemas can be challenging, especially in newborns, in ultra-short HSCR and in those with TCA, in whom the colon can appear normal in caliber [13,14]. Thus, intraoperative confirmation based on FSB of the presence of ganglion cells and non-hypertrophied nerve fibers is needed for determining of the level of resection [5,15]. It is common for many pediatric surgeons to also resect the dilated segment to circumvent challenges in performing the anastomosis and address issues related to the dysmotility of the dilated segment. However, examining samples from FSB carries the risk of technical errors, primarily related to tissue handling and freezing artifacts, leading to increased costs and time. Additionally, FSB may not always be available, especially in resource-limited settings. Most studies have confirmed that the length of the TZ varies but is typically ≤5 cm in rectosigmoid HSCR [16,17]. In rare cases, the TZ can be longer than 15 cm, exhibiting hypoganglionosis in the longitudinal antimesenteric aspect due to an irregular distribution of ganglion cells in the TZ [18].

In our study, the level of resection in the NGS was determined solely based on macroscopic assessments intraoperatively. The accuracy of this evaluation was confirmed postoperatively by verifying the presence of ganglion cells and non-hypertrophied nerve fibers in all resections at least 5 cm distally from the proximal resection margins, 5 cm above the TZ. This conclusion aligns with findings from previous research [4,16]. Predicting the level of resection without HP confirmation from the FSB raises concerns about safety. Thakkar et al. highlighted that not all surgeons wait for a frozen section review of the proximal donut before completing the pull-through procedure. This suggests that there may be variability in surgical practices regarding the reliance on FSB for confirmation during the procedure [19]. In a study by Beltman et al., resection was performed in the TZ in 10 out of 123 cases. In these cases, the surgeon’s intraoperative judgment was used to determine the correct level of resection, considering the dilated segment above the TZ as the level where healthy bowel was expected to be encountered for the first FSB [20]. Also, in a study by Saad et al., resection was performed 5–10 cm above the TZ without intraoperative determination of the level of resection using FSB [21]. In our study, for cases with the AGS located up to the distal half of the sigmoid colon, resection was performed 5–10 cm above the visualized TZ. Postoperative microscopic examinations of the resections confirmed that the macroscopic assessment determined the level of resection in the NGS. Some hospitals continue to rely on contrast enemas for the preoperative determination of AGS and TZ due to the unavailability of frozen section facilities [9,10]. In our study, contrast enemas served not only for preoperative planning but also to exclude cases with long-segment HSCR and TCA, in which a two-staged procedure with an intraoperative full thickness biopsy (FTB) was performed. 

None of the cases in our study were ultra-short HSCR, and only three cases were excluded due to TCA. In some instances, pediatric surgeons, when clinical and imaging signs are highly indicative of HSCR, initiate the operative procedure without prior histopathological confirmation by performing the first histopathological confirmation from the transrectal biopsy, performed as an FSB [19,21], and then proceed with the operation planned based on the contrast enema findings. The procedure continues until at least 5 cm above the last confirmation of ganglion cells and non-hypertrophied nerve fibers, confirmed from FSB, where they then perform the bowel resection [8]. The primary intraoperative challenge lies in determining the level of resection above the TZ to avoid complications related to retaining the TZ. Recommendations have varied widely regarding the resection length over the transitional zone, ranging from 2 cm to 15 cm [4]. Schäppi et al. recommend that the proximal resection margin should be at least 2–3 cm of colon proximal to the first biopsy showing a normal ganglion density [14]. White and Langer recommended excising more than 2 cm proximal to the most distant ganglionic biopsy to avoid a transition zone pulled through (TZPT) [22,23,24]. Also, Smith, C. and Kapur, R.P. et al. proposed that the level of resection should be performed at least 5 cm proximal to the TZ [4]. In an article by Jeffry, A. and Marc, L. the TZ is described to measure between 1 and 10 cm. They recommend performing an anastomosis 3–10 cm above the biopsy site with normal ganglion cells [18]. Tomuschat C. et suggest that performing the colo-anal anastomosis 5–10 cm proximal to the most distant ganglionic biopsy is safe [17]. Georgeson recommended that a 10–15 cm margin of ganglionic colon should be resected prior to completing the anastomosis [25]. 

The presence of the TZ in the anastomosis is associated with constipation and a higher incidence of HAEC [26]. Conversely, despite undergoing complete resection of the AGS and TZ, some patients still suffer from severe constipation or episodes of HAEC, while patients with a TZPT have a higher risk for constipation and HAEC [27]. However, De la Torre et al. stated that there was no difference in these complications between those with and without TZPT [28]. In our research, among the 60 cases operated for Hirschsprung’s disease (HSCR), signs of constipation were identified in 13 patients after the operation due to colon hypomotility, which were managed with conservative therapy. During the two-decade follow-up of these cases, HAEC caused one hospitalization in 17 cases, with two cases experiencing multiple episodes within two years (three and four times, respectively). This indicates the need for repeated contrast enemas and transrectal biopsies in one case, despite the confirmation of the presence of a NGS from their resection. From the FTHB performed 2 and 4 cm above the dentate line, it was observed that rare ganglion cells were found (hypoganglionosis) in the sample taken at the second centimeter above the dentate line, while a normally ganglionated segment was confirmed in the fourth centimeter. This finding was reconfirmed via IHC re-examination, which showed the presence of a normally ganglionated segment up to the seventh centimeter distally from the resected margin. The hypoganglionosis observed at this level may have been due to possible partial ischemia at the level of the anastomosis, a phenomenon reported in the literature as a cause of hypoganglionosis and aganglionosis [29,30]. In the same patient, post-operative stenosis developed, requiring dilation treatment for three months. This stenosis may also have been a consequence of the ischemia of the anastomosis. 

While determining the normoganglionic “safe zone” through FSB is considered safer than relying solely on the surgeon’s intraoperative judgment, it is important to acknowledge the considerable degree of subjectivity in correctly identifying the hypoganglionic zone from the normal ganglionic one, as well as the duration of anesthesia until receiving the result from the FSB. In our research involving 60 cases operated without FSB, none underwent FSB, reducing the duration of anesthesia and avoiding additional expenses associated with involving the pathologist, making it a less costly and more efficient approach that reduces the number of FSB procedures required. The comparison of macroscopic and microscopic results in our research showed a significant correlation, suggesting that macroscopic assessment can reliably predict the presence of NGS in cases with short-segment HSCR. However, the accuracy of macroscopic evaluations has been shown to be much higher in experienced hands [9,10,20]. Additionally, it can be challenging to identify the TZ in neonates, and in some cases, children under the age of three months, even for experienced pediatric surgeons, as demonstrated by contrast enema studies [1,31,32]. 

We conducted a thorough literature review and found that no previous research has utilized a comparison between microscopic and macroscopic evaluations for decision-making regarding the level of resection during definitive operation in HSCR. In our study, the sensitivity of the macroscopic evaluations was 100%. However, we believe this result may be attributed to several factors. Firstly, all cases in our study were above 9 months of age, and in each case, the AGS was located between the sixth centimeter above the dentate line and the lower third of the descending colon. Secondly, our surgical approach involved resecting 5–10 cm above the TZ in cases where the AGS was located until the lower half of the sigmoid colon and had a less dilated segment, and 10–15 cm in cases from the proximal half of the sigmoid colon until the lower third of the descending colon, accompanied by a more pronounced dilated segment. We acknowledge that the TZ may be challenging to identify intraoperatively, especially in ultra-short-segment cases and neonates, as the TZ is not well differentiated in these situations. Our study primarily focused on the accuracy of intraoperative predictions of the resection level in the normally ganglionated segment using morphologic changes in the bowel segments in short-segment HSCR. We believe that this method is less accurate in long-segment HSCR, since the TZ is much longer, and it has also been found to have less concordance with contrast enemas. Furthermore, we emphasize the need for further multicenter studies with a larger sample size to validate and confirm our findings.

## 5. Limitations

The surgeon’s experience in assessing the TZ and NGS, as well as subgroups of patients such as those aged less than 3 months and those with ultra-short HSCR, are considered possible limitations for macroscopic assessments in predicting the level of resection in NGS. It should be tested by other centers to strengthen the validity of this method, which would lower cost expenses and reduce additional time during operations, especially in short-segment HSCR, which includes most of Hirschsprung’s disease cases.

## 6. Conclusions

Despite the limitations, macroscopic evaluation is an alternative to FSB in predicting the level of bowel resection. We believe that it can be an accurate method when used by experienced surgeons in short-segment HSCR patients older than 6 months. It should be supplemented by FSB when the macroscopic assessment is inconclusive. It is crucial to involve the parents/legal guardians in the decision-making process due to the potential implications of proceeding without frozen sections.

## Figures and Tables

**Table 1:** Length of the normoganglionic segment above the transition zone in resections.

Length of Normoganglionic Segment in Resections	Number of Cases
5 cm	7
7 cm	18
10 cm	23
15 cm	12

**Table 2:** Evaluation of macroscopic and microscopic concordance.

Evaluation	Macroscopic Evaluation: Normal	Macroscopic Evaluation: Abnormal
Microscopic evaluation: normal	True Positives—60	False positives—0
Microscopic evaluation: abnormal	False negatives—0	True negatives—0

**Table 3:** Comparison of accuracy in second and fifth cm above transition zone.

Evaluation	True Positive	Accuracy
Macroscopic	17	28.33%
Microscopic	60	100%

## Data Availability

Data Availability Statements are available in section “MDPI Research Data Policies” at https://www.mdpi.com/ethics.

## Abbreviations

AGS	Aganglionic segment
FN	False negatives
FP	False positives
FSB	Frozen-section biopsy
FTB	Full-thickness biopsy
HE	Hematoxylin and eosin
HSCR	Hirschsprung’s disease
HAEC	Hirschsprung-associated enterocolitis
IHC	Immunohistochemistry
NGS	Normoganglionic segment
Ss-HSCR	Short-segment Hirschsprung’s disease
TCA	Total colon aganglionosis
TP	True positives
TN	True negatives
TZ	Transition zone
TZPT	Transition zone pull-through
TEPT	Trans anal endorectal pull-through

## References

[1] Jamieson D.H., Dundas S.E., Al Belushi S., Cooper M., Blair G.K. (2004). Does the transition zone reliably delineate aganglionic bowel in Hirschsprung’s disease?. Pediatr. Radiol..

[2] Kapur R.P., Kennedy A.J. (2012). Transitional zone pull through: Surgical pathology considerations. Semin. Pediatr. Surg..

[3] Frongia G., Günther P., Schenk J.P., Strube K., Kessler M., Mehrabi A., Romero P. (2016). Contrast Enema for Hirschsprung Disease Investigation: Diagnostic Accuracy and Validity for Subsequent Diagnostic and Surgical Planning. Eur. J. Pediatr. Surg..

[4] Smith C., Ambartsumyan L., Kapur R.P. (2020). Surgery, Surgical Pathology, and Postoperative Management of Patients with Hirschsprung Disease. Pediatr. Dev. Pathol..

[5] Maia D.M. (2000). The reliability of frozen-section diagnosis in the pathologic evaluation of Hirschsprung’s disease. Am. J. Surg Pathol..

[6] Kapur R.P., Arnold M.A., Conces M.R., Ambartsumyan L., Avansino J., Levitt M., Wood R., Mast K.J. (2019). Remodeling of Rectal Innervation After Pullthrough Surgery for Hirschsprung Disease: Relevance to Criteria for the Determination of Retained Transition Zone. Pediatr. Dev. Pathol..

[7] Teeraratkul S. (2003). Transanal one-stage endorectal pull-through for Hirschsprung’s disease in infants and children. J. Pediatr. Surg..

[8] Shehata S. Avoiding Complications in Hirschsprung’s Disease, by Prof Sameh Shehata, Hosted by Ankara University—YouTube. https://www.youtube.com/watch?v=iH5-G9OCUqw.

[9] Negash S., Getachew H., Tamirat D., Mammo T.N. (2022). Hirschsprung disease managed with one-stage transanal endorectal pullthrough in a low-resource setting without frozen section. BMC Surg..

[10] Choudhury K.M., Jafor A., Ahmed S. (2014). Transanal Endorectal Pullthrough for Hirschsprung’s Disease Without Frozen-Section Biopsy Facility. BIRDEM Med. J..

[11] Hall N.J., Rahman A., Morini F., Prato A.P., Friedmacher F., Koivusalo A., van Heurn E., Pierro A., Zani A. (2019). European Paediatric Surgeons’ Association Survey on the Management of Pediatric Appendicitis. Eur. J. Pediatr. Surg..

[12] Proctor M., Traubici J., Langer J., Gibbs D., Ein S., Daneman A., Kim P. (2003). Correlation between radiographic transition zone and level of aganglionosis in Hirschsprung’s disease: Implications for surgical approach. J. Pediatr. Surg..

[13] Stranzinger E., DiPietro M.A., Teitelbaum D.H., Strouse P.J. (2008). Imaging of total colonic Hirschsprung disease. Pediatr. Radiol..

[14] Schäppi M., Staiano A., Milla P., Smith V., Dias J., Heuschkel R., Husby S., Mearin M., Papadopoulou A., Ruemmele F. (2013). A Practical Guide for the Diagnosis of Primary Enteric Nervous System Disorders. J. Pediatr. Gastroenterol. Nutr..

[15] Langer J.C., Durrant A.C., de la Torre L., Teitelbaum D.H., Minkes R.K., Caty M.G., Barbara E.W., Jose J.S., Shinjiro H., Craig A. (2003). One-Stage Transanal Soave Pullthrough for Hirschsprung Disease: A Multicenter Experience with 141 Children. Trans. Meet Am. Surg Assoc..

[16] Kapur R.P., Kennedy A.J. (2013). Histopathologic Delineation of the Transition Zone in Short-Segment Hirschsprung Disease. Pediatr. Dev. Pathol..

[17] Tomuschat C., Mietzsch S., Dwertmann-Rico S., Clauditz T., Schaefer H., Reinshagen K. (2022). The Length of the Transition Zone in Patients with Rectosigmoid Hirschsprung Disease. Children.

[18] Jeffrey R.A., Marc L.A. (2016). Hirschsprung Disease. Fundam Pediatr Surgery.

[19] Thakkar H.S., Blackburn S., Curry J., De Coppi P., Giuliani S., Sebire N., Cross K. (2020). Variability of the transition zone length in Hirschsprung disease. J. Pediatr. Surg..

[20] Beltman L., Shirinskiy I., Donner N., Backes M., Benninga M., Roelofs J., van der Voorn P., van Schuppen J., Oosterlaan J., van Heurn E. (2023). Determining the Correct Resection Level in Patients with Hirschsprung Disease Using Contrast Enema and Full Thickness Biopsies: Can the Diagnostic Accuracy be Improved by Examining Submucosal Nerve Fiber Thickness?. J. Pediatr. Surg..

[21] Saad S.A., Elseed M.M.G., AbouZeid A.A., Ibrahim E.A., Radwan A.B., Hay S.A., El-Behery M.M. (2020). Histopathological perspective of the pulled-through colon in disease: On clinical outcome. J. Pediatr. Surg..

[22] White F.V., Langer J.C. (2000). Circumferential Distribution of Ganglion Cells in the Transition Zone of Children with Hirschsprung Disease. Pediatr. Dev. Pathol..

[23] Coyle D., O’Donnell A.M., Tomuschat C., Gillick J., Puri P. (2019). The Extent of the Transition Zone in Hirschsprung Disease. J. Pediatr. Surg..

[24] Kapur R.P. (2016). Histology of the Transition Zone in Hirschsprung Disease. Am. J. Surg. Pathol..

[25] Georgeson K.E. (2002). Laparoscopic-Assisted Pull-Through for Hirschsprung’s Disease. Semin. Pediatr. Surg..

[26] Langer J.C., Rollins M.D., Levitt M., Gosain A., de la Torre L., Kapur R.P., Cowles R.A., Horton J., Rothstein D.H., On behalf of the American Pediatric Surgical Association Hirschsprung Disease Interest Group (2017). Guidelines for the management of postoperative obstructive symptoms in children with Hirschsprung disease. Pediatr. Surg. Int..

[27] Levitt M.A., Dickie B., Peña A. (2010). Evaluation and treatment of the patient with Hirschsprung disease who is not doing well after a pull-through procedure. Semin. Pediatr. Surg..

[28] Wehrli L.A., Reppucci M.L., Stevens J., Arnold M., Lovell M., Zornoza M., Bischoff A., De la Torre L. (2023). Should we perform a Hirschsprung redo pull-through on patients with retained transition zone?. J. Pediatr. Surg. Open.

[29] Earlam R.J. (1972). A vascular cause for aganglionic bowel. Am. J. Dig. Dis..

[30] Taguchi T., Suita S., Hirata Y., Hirose R., Yamada T., Toyohara T. (1994). Abnormally shaped arteries in the intestine of Children with Hirschsprung’s disease: Etiological Considerations Relating to Ischemic Theory. J. Pediatr. Gastroenterol. Nutr..

[31] O’Donovan A.N., Habra G., Somers S., Malone D.E., Rees A., Winthrop A.L., O’Donovan G.H.A.N., Martin L.C., Merkle E.M., Thompson W.M. (1996). Diagnosis of Hirschsprung’s disease. Am. J. Roentgenol..

[32] Sahu R.K., Kothari S., Rahaman S.R., Chattopadhyay A., Dasgupta S., Sen S. (2017). Evaluation of suspicious Hirschsprung disease in children using radiologic investigation method: A prospective observational study. Int. Surg. J..

